# Risk of Premature Ovarian Insufficiency after Human Papilloma Virus Vaccination: A PRISMA Systematic Review and Meta-Analysis of Current Evidence

**DOI:** 10.3390/vaccines11010140

**Published:** 2023-01-09

**Authors:** Marco Torella, Maria Maddalena Marrapodi, Carlo Ronsini, Alessandro Ferdinando Ruffolo, Andrea Braga, Matteo Frigerio, Emanuele Amabile, Maria Giovanna Vastarella, Francesca Rossi, Gaetano Riemma

**Affiliations:** 1 Department of Woman, Child and General and Specialized Surgery, University of Campania “Luigi Vanvitelli”, 80128 Naples, Italy; 2 Department of Obstetrics and Gynecology, IRCSS San Raffaele Scientific Institute, 20132 Milan, Italy; 3 Department of Obstetrics and Gynecology, EOC, Beata Vergine Hospital, 6850 Mendrisio, Switzerland; 4 ASST Monza, Ospedale San Gerardo, 20900 Monza, Italy

**Keywords:** human papilloma virus: HPV, vaccine: premature ovarian failure, premature ovarian insufficiency, infertility

## Abstract

(1) Background: Premature ovarian insufficiency (POI) has been linked to human papilloma virus (HPV) vaccination in small case-reports. The aim of this meta-analysis was to evaluate the risk of POI after HPV vaccination. (2) Methods: Electronic searches in MEDLINE Scopus, LILACS, ClinicalTrials.gov, EMBASE, PROSPERO, Cochrane CENTRAL and other registries were searched from inception to September 2022. Inclusion criteria were cohort studies of female children or adolescents vaccinated with quadrivalent (4vHPV), bivalent (2vHPV) or 9-valent (9vHPV) vaccine and compared to unvaccinated, other HPV vaccine, or vaccinated with other childhood vaccine girls. Primary outcome was risk of POI after vaccination. (3) Results: Four studies, including 1,253,758 patients, were included. Overall, there was no significant risk for POI between 4vHPV and controls (unvaccinated or other vaccines) (RR 0.47 (95% CI 0.14 to 1.59) I^2^ = 75%), or unvaccinated only controls (RR 0.75 (95% CI 0.22 to 2.49) I^2^ = 26%). One study reported a significant reduction of POI risk for 4vHPV relative to the other childhood vaccinations (RR 0.03 (95% CI 0.00 to 0.21)); meanwhile, one study showed no increased risk with 4vHPV relative to 2vHPV and 9vHPV (RR 0.93 (95% CI 0.33 to 2.64)). (4) Conclusions: 4vHPV vaccination does not seem to increase risk of POI relative to unvaccinated people or other childhood vaccines. No difference was seen with 4vHPV vaccine relative to 2vHPV and 9vHPV. Moreover, the risk of POI after HPV vaccination is relatable to worldwide incidence, giving reassurance about safety.

## 1. Introduction

Human papillomavirus (HPV) is the most common sexually transmitted infection in the United States [1]. In female patients, the administration of the HPV vaccine can successfully prevent cervical cancer, since ongoing infection with high-risk HPV strains is strongly linked to its development [2]. HPV vaccinations have been authorized and advised for use in teenage girls since 2006. Currently, three prophylactic HPV vaccines, HPV bivalent recombinant vaccine (2vHPV), HPV quadrivalent recombinant vaccine (4vHPV), and HPV 9-valent recombinant vaccination (9vHPV), are available worldwide [3]. 

In comparison to other recommended adolescent immunizations, including tetanus toxoid, reduced diphtheria toxoid, acellular pertussis, adsorbed (Tdap), and meningococcal conjugate (MenACWY), HPV vaccination rates have lagged [4].

In fact, false safety worries have prevented many nations from accepting routine HPV vaccination [5].

After case studies detailing the onset of premature ovarian insufficiency (POI), also known as premature ovarian failure or premature menopause, in six young women between the ages of 13 and 21 within a year of vaccination were published, there was an increased concern about infertility following HPV vaccination [6].

Menstrual disruption (amenorrhea or oligomenorrhea), elevated gonadotrophins, and decreased estradiol levels are the hallmarks of POI, a clinical disease caused by loss of ovarian function before the age of 40 [7]. Few chances of spontaneous pregnancy exist in women with POI, and no therapies have proven effective in boosting ovarian activity and rates of natural conception [8].

The presence of updated guidelines, the improvement of diagnostic capabilities and the use of additional serum and genetic markers have increased the POI detection rate, the global pooled prevalence of which is estimated to be about 3.7% [9,10]. Prevalence is higher in low or middle relative to high income countries [9]. 

Therefore, POI is a rare outcome, and an evaluation of its association with HPV vaccination necessitates large observational data sources. 

Published adverse reactions about HPV vaccines were mainly available from clinical trials, which might not reflect the full safety characteristics of HPV vaccines because of strict trial design, relatively small sample size and short duration of follow-up [11]. For this reason, the most useful data sources for identifying and discovering new or rare adverse reactions are vaccination adverse reaction registries, with all the limitations that are related to their nature [12]. 

To this purpose, the aim of this systematic review and meta-analysis was to analyze a plausible link between POI and HPV vaccination and, therefore, provide reassurance about its safety.

## 2. Materials and Methods

This meta-analysis was performed following the Preferred Reporting Items for Systematic reviews and Meta-Analyses (PRISMA) [13]. The study protocol was built a priori, and it carefully described the literature search and reporting, inclusion and exclusion of articles, data analysis, and statistical procedures. 

### 2.1. Data Sources and Search Strategy 

Electronic databases including EMBASE, MEDLINE (accessed through PubMed), Scopus, Scielo.br and LILACS were searched with the use of the following keywords and Medical Subject Heading (MeSH) terms: “human papillomavirus” or “HPV” and “vaccination” or “vaccine” and “premature ovarian failure” or “premature ovarian insufficiency” or “amenorrhea” without any date restriction to September 2022. Searches were also conducted on CINAHL, PsycINFO, and AMED to find other relevant papers and avoid publication bias. To seek for additional trials, Clinicaltrials.gov, Cochrane Central Register of Controlled Trials and World Health Organization International Clinical Trials Registry Platform (ICTRP) were also searched. In addition, the gray literature (NTIS, PsycEXTRA) was evaluated to find abstracts of international and national conferences. We also checked the reference lists of the included papers to add further studies not captured during the original search. 

There was no language or geographic location restriction applied. Commentaries, letters to the editors, editorials, meta-analyses and review articles were excluded from the search. 

### 2.2. Study Selection Criteria and Data Extraction

The inclusion criteria were any randomized, prospective or retrospective studies that included female children and adolescents vaccinated for HPV with 2vHPV, 4vHPV or 9vHPV and had a control group consisting of unvaccinated children or vaccinated for other pathogens of infancy (Tdap, MenACWY, tetanus toxoid, hemophilus influenzae, seasonal flu viruses).

The abstraction forms were designed specifically for this meta-analysis. The key characteristics that were recorded included the following: the patient descriptors, study duration, setting, details of HPV vaccine, features of the control group, outcomes evaluated, mean follow-up length, results and quality of evidence analysis. 

All the abstracts were screened and classified by two authors (G.R., M.T.) independently. The agreement for plausible relevance was reached by consensus; the same two authors carried out a full text assessment of the selected papers and independently extracted marked data on the study characteristics and the outcomes of interest. All the inconsistencies were discussed by the reviewers and a consensus was reached by consulting a third author (M.M.M.). If necessary, unpublished data were obtained by direct contact with the authors of the original studies whenever the study methodology indicated that other outcome data were recorded. 

### 2.3. Main Outcome Measures 

The primary outcome of this meta-analysis was the risk of POI after the 4vHPV vaccination. Secondary endpoints were POI incidence according to the HPV vaccine subtype and differences in risk ratio relative to the common childhood vaccinations.

The diagnosis of POI was made in the included studies according to the American College of Obstetricians and Gynecologists (ACOG) guidelines for the diagnosis of POI. Such criteria include the presence of menstrual irregularity for at least 3 months and elevated follicle-stimulating hormone (FSH) in the postmenopausal range and low estradiol levels on two separate samplings. Other diagnostic tests used in available papers to establish, exclude or refine the diagnosis include karyotyping, adrenal antibody sampling and serum Antimullerian hormone levels.

### 2.4. Assessment of Risk of Bias 

The Newcastle–Ottawa Scale criteria were used to assess the risk of bias in each of the included research studies [14]. These criteria state that the selection and comparability of study groups, as well as the determination of the outcome of interest, form the basis for the study’s evaluation. Evaluation of the exposed cohort’s representativeness, choice of the non-exposed cohort, determination of exposure, and proof that the desired outcome was unlikely to occur spontaneously at the beginning of the study are the criteria used to select a study. By examining the equivalency of cohorts based on the design or analysis, the comparability of research is evaluated [14].

Additionally, the effectiveness of the exposure is assessed based on the methods used to gauge follow-up time, quality and outcome of interest. A study may receive a maximum of one star on the Newcastle–Ottawa Scale for each numbered item in the Selection and Outcome categories. For comparability, a maximum of two stars may be assigned. According to the Newcastle–Ottawa Scale criteria, a maximum score of nine could be assigned [14].

### 2.5. Data Analysis

To analyze the data, Review Manager 5.3 (The Nordic Cochrane Centre 2014) was used. After using Der Simonian and Laird’s random-effects model, the summary measures were presented as a risk ratio (RR) or mean difference with a 95% confidence interval (CI). To overcome potential heterogeneity, a Higgins I^2^ index greater than 0% was considered, whereas 25%, 50% and 75% were considered cut-offs for low, intermediate, and high heterogeneity. Subgroup analysis was performed to investigate the influence of data from adverse reaction registries to the outcome of interest. The potential publication bias was investigated using the visual evaluation of the funnel plot and the Egger test. A *p* value less than 0.05 was considered statistically significant. 

## 3. Results

Initially, 82 studies were identified through database search. Of those, 11 were removed as duplicates. After title and abstract screening, 64 papers were removed as editorials and letters to editor (12 records), review articles (27 records), or out of topic (25 records). 

**Figure 1:**
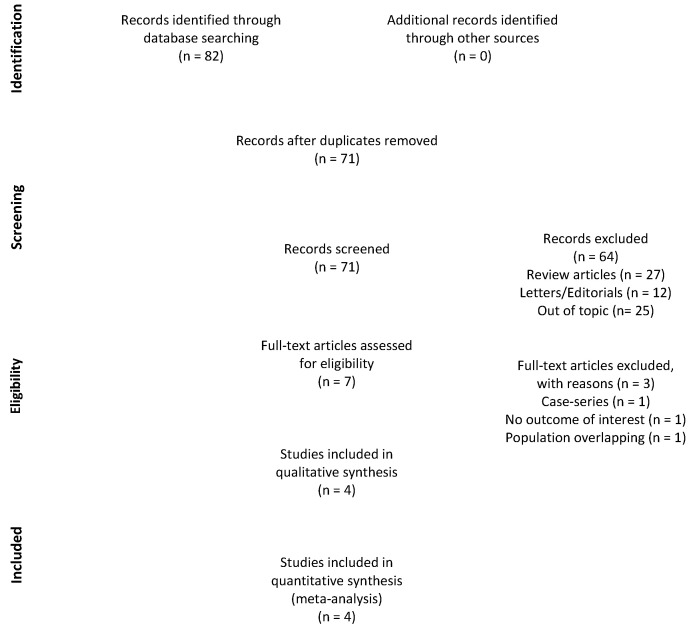
Flowchart of studies included in systematic review and meta-analysis.

Seven studies were selected, of which one was removed for being a case-series, one for the absence of the outcome of interest and one for population overlapping. Four studies with 1,253,758 participants were included in quantitative synthesis and meta-analysis [4,15,16,17] (Figure 1). 

One study was a retrospective analysis of nationwide cohorts of vaccinated vs. unvaccinated female children, including all the female children who joined the national HPV vaccination plan [15]. Another study analyzed adverse events of special interest for seeking HPV cases [17]. Another paper focused its aim by evaluating POI among adverse events registries related to HPV vaccination [16]. Another paper compared the 4vHPV vaccine to other childhood vaccination retrieving data from passive surveillance [4] (Table 1).

Inclusion and exclusion criteria for retrieved studies are depicted in Table 2.

### 3.1. Quality Assessment

All examined trials reported high scores using the Newcastle–Ottawa Scale criteria, with values ranging from a minimum of 6 to a maximum of 8. Based on controls for age as the primary element and BMI as an extra factor, the comparability of cohorts achieved its highest level. Table S1 in the Supplementary Materials illustrates the thorough point-by-point evaluation.

### 3.2. Synthesis of Results

**Figure 2:**
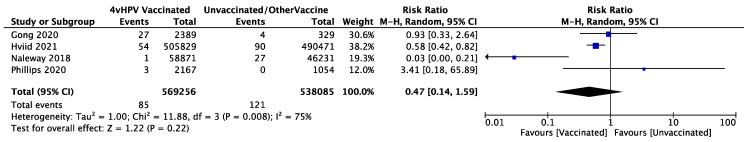
Risk of POI with 4vHPV vaccination relative to other or no vaccination [4,15,16,17].

Considering all the data provided for the 4vHPV vaccine compared to other vaccines or unvaccinated people, there was no significant risk for POI between treatment and controls (RR 0.47 (95% CI 0.14 to 1.59) I^2^ = 75%) (Figure 2).

**Figure 3:**
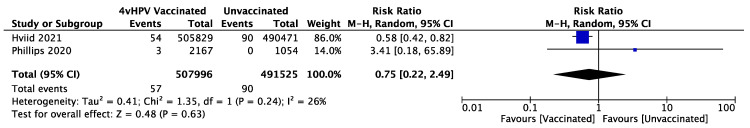
Risk of POI with 4vHPV vaccination relative to no vaccination [15,16].

Two studies evaluated the differences between HPV vaccination with 4vHPV and no vaccination; the incidence of POI did not differ between groups (RR 0.75 (95% CI 0.22 to 2.49) I^2^ = 26%) (Figure 3).

Relative to 2vHPV and 9vHPV, a similar risk for POI was seen with the 4vHPV vaccine in the analysis conducted by Gong et al. [17] (RR 0.93 (95% CI 0.33 to 2.64)). 

On the contrary, Naleway et al. [4] showed a marked reduction of POI risk for 4vHPV relative to the other childhood vaccinations (RR 0.03 (95% CI 0.00 to 0.21)).

### 3.3. Subgroup Analysis

To explain the between-studies heterogeneity, we conducted a subgroup analysis that excluded papers that retrieved data from national passive surveillance registries [4,16]. In this case, there was a reduced risk of POI in women subjected to HPV vaccine relative to unvaccinated women, with no residual heterogeneity remaining (RR 0.61 (95% CI 0.44 to 0.84) I^2^ = 0%).

## 4. Discussion

This systematic review and meta-analysis showed that HPV vaccination could be safely administered to female children since reassuring data about the risk of POI, which was similar in unvaccinated girls, were obtained.

Studying POI as a vaccine adverse event is challenging for many reasons. The most important relies on the fact that the time from symptom onset to diagnosis with POI may be variable or long [18]. 

On the connection between HPV vaccination and POI, little research has been conducted. A case study on a 16-year-old Australian girl served as the inspiration for the suggested association. Menarche happened at the age of 13 but was then followed by oligomenorrhea and amenorrhea for 17 months. Menstrual abnormalities were reported to begin after the girl received her HPV immunization. Following this case report, the same authors of the original report submitted two additional cases as well as a case series of three other girls, including two sisters, who had all had HPV vaccinations prior to the diagnosis of POI [6,19]. 

Numerous explanations for the plausible relationship between POI and HPV vaccines have been put out, including autoimmune reactions to the aluminum adjuvant in the vaccine and purported ovarian toxic effects related to polysorbate 80, which is used as an excipient in the vaccine [20]. 

Although a precise cause for POI has not been shown, postvaccination autoimmunity is theoretically feasible, and polysorbate 80 exposure levels from vaccine exposure are negligible in comparison to hazardous levels. Therefore, such risk should be considered theoretically unreasonable [20,21].

Similarly, other vaccines contain polysorbate 80, such as vaccines against rotavirus, pneumococcal and meningococcal diseases, diphtheria, tetanus, pertussis, influenza, hepatitis A and B, and poliomyelitis, with no increased risk of POI [12,21]. 

In addition, a recent study ascertained the safety of HPV vaccination on reproductive and pregnancy outcomes of women undergoing in vitro fertilization. Demir et al. showed that, compared to unvaccinated women, HPV vaccinated patients had similar rates of retrieved oocytes and mature oocytes with unsignificant differences in implantation, clinical and ongoing pregnancy rates [22].

On the contrary, Tatang et al. reported a potential safety signal regarding POI after HPV vaccination POI, analyzing the Vaccine Adverse Event Reporting System (VAERS) database for POI cases [23]. However, they stated that if that signal was confirmed by additional epidemiological studies, such risk would be smaller if compared to the lifetime risk of cervical cancer. Moreover, it should be noted that, as for other studies based on passive surveillance, the study might be subjected to reporting bias and its results should be interpreted with caution [23].

This systematic review shows several points of strength. First, it is the first study incorporating data from different groups of females subjected to HPV vaccine in a meta-analysis. Second, despite the limited number of studies, the overall number of participants should be considered enough to guarantee the robustness of the findings. Lastly, all the data used for the analysis come from single cohorts, without overlapping of data from studies that evaluated the same cohorts of women among national registries.

However, several limitations should be accounted to this quantitative synthesis. First, the small number of included studies, which is a main limitation to the overall conclusions of the study. Secondly, the results may be affected by population bias due to the retrospective design of included papers. Third, half of the included papers were analyses of vaccine adverse reaction registries due to passive surveillance; since these databases accept any kind of report by physicians, without checking the plausibility of the source, such issue could lead to between-studies heterogeneity and misrepresent the real evidence. 

In fact, the quality and thoroughness of POI case reports varied, with most of them lacking details on levels of estrogen and follicle-stimulating hormone. The reported HPV vaccine and POI events may or may not represent real instances, with POI serving as the correct diagnosis, according to incomplete data.

For this reason, we performed a subgroup analysis that excluded this category of reports, lowering the heterogeneity to absent and further confirming our findings.

An additional limitation should be accounted to the differences in POI among the countries, which might reduce the generalization of the findings.

## 5. Conclusions

HPV vaccination, in its commercially available forms, could be safely administered to female children and adolescents with no increased risk for POI relative to unvaccinated people or other childhood vaccines. Although extremely rare, most POI cases were seen with similar incidence using the 4vHPV vaccine relative to 2vHPV and 9vHPV. Moreover, the risk of developing POI after 4vHPV is comparable to POI incidence in the overall population, providing reassurance about its safety.

## Figures and Tables

**Table 1:** Main characteristics of studies included in quantitative synthesis and meta-analysis.

Study, Year	Design	Location	Duration	Population	Intervention	Control	Outcomes	Sample Size
Gong, 2020 [17]	Retrospective cohort study	China	2006–2018	9–18 years old females	4vHPV	2vHPV 9vHPV	Diagnoses of POI and related symptoms	418
Hviid, 2021 [15]	Retrospective cohort study	Denmark	2007–2016	Girls aged between 12–15	4vHPV vaccination	Unvaccinated girls	Diagnoses of POI	1,051,041
Naleway, 2018 [4]	Retrospective cohort study	USA	2006–2014	NA	HPV vaccinated girls	Other vaccine (tetanus toxoid, Tdap, influenza)	Risk of POI	199,078
Phillips, 2020 [16]	Retrospective cohort study	Australia	2007–2017	12–13 years old male and female children	4vHPV vaccination	Unvaccinated children	Adverse events of special interest following 4vHPV vaccination	3221

NA: not available.

**Table 2:** Inclusion and exclusion criteria for included studies.

Study, Year	Inclusion Criteria	Exclusion Criteria
Gong, 2020 [17]	Women inserted in the HPV- vaccine-related adverse events	NA
Hviid, 2021 [15]	Women added in a register-based research study of all Danish-born girls and women aged 11 to 34 years during 2007 to 2016.	NA
Naleway, 2018 [4]	Women with premature ovarian insufficiency from a non-genetic cause	Premature ovarian insufficiency from other known causes (fragile X Syndrome, Turner Syndrome or other chromosomal disorders)
Phillips, 2020 [16]	Women with a severe adverse event following 4vHPV vaccination	Women with an adverse event following 9vHPV or 2vHPV HPV vaccines.

NA: not available.

## Data Availability

The data presented in this study are available on request from the corresponding author.

## Supplementary Materials

The following supporting information can be downloaded at: https://www.mdpi.com/article/10.3390/vaccines11010140/s1, Table S1: Detailed risk of bias assessment using Newcastle–Ottawa Scale criteria.
