# Supplementary material for: Risk of Premature Ovarian Insufficiency after Human Papilloma Virus Vaccination: A PRISMA Systematic Review and Meta-Analysis of Current Evidence
Source: Vaccines (Basel). 2023 Jan 9;11(1):140. doi: 10.3390/vaccines11010140 (PMC9866915; doi:10.3390/vaccines11010140)
Supplement: Supplementary file 1 [file vaccines-11-00140-s001.zip › vaccines-2087899-supplementary.pdf]

Table S1. Quality scores of the studies included in the meta-analysis, assessed by the Newcastle-Ottawa scale.

		Selection				Comparability [a]	Outcome			Overall quality
**Author**	**Year**	**Representativeness of the exposed cohort**	**Selection of the non-exposed cohort**	**Ascertainment of exposure**	**Demonstration that the outcome of interest was not present at the start of the study**	**Comparability of cohorts on the basis of the design or analysis**	**Assessment of the outcome**	**Was follow-up enough for outcomes to occur**	**Adequacy of follow-up of cohorts**	
Hviid	2021	*	*	*	*	*	*	*	*	**8**
Phillips	2020	*	*	0	*	*	0	*	*	**6**
Gong	2020	*	*	*	*	*	*	*	*	**8**
Naleway	2018	*	*	0	*	*	0	*	*	**6**

Newcastle-Ottawa scale for assessment of quality of included studies - cohort studies (each asterisk represents if individual criterion within the subsection was fulfilled). [a] Comparability of cohorts: for the most important factor: study controls adjusted for age; additional factor: study controls for BMI
